# An Improved Fire and Smoke Detection Method Based on YOLOv8n for Smart Factories

**DOI:** 10.3390/s24154786

**Published:** 2024-07-24

**Authors:** Ziyang Zhang, Lingye Tan, Tiong Lee Kong Robert

**Affiliations:** School of Civil & Environmental Engineering, Nanyang Technological University, Singapore 639798, Singapore; ziyang004@e.ntu.edu.sg (Z.Z.); lingye001@e.ntu.edu.sg (L.T.)

**Keywords:** factory fire and smoke detection, YOLOv8n, ConvNextV2, RepBlock, SimConv, MPDIoU

## Abstract

Factories play a crucial role in economic and social development. However, fire disasters in factories greatly threaten both human lives and properties. Previous studies about fire detection using deep learning mostly focused on wildfire detection and ignored the fires that happened in factories. In addition, lots of studies focus on fire detection, while smoke, the important derivative of a fire disaster, is not detected by such algorithms. To better help smart factories monitor fire disasters, this paper proposes an improved fire and smoke detection method based on YOLOv8n. To ensure the quality of the algorithm and training process, a self-made dataset including more than 5000 images and their corresponding labels is created. Then, nine advanced algorithms are selected and tested on the dataset. YOLOv8n exhibits the best detection results in terms of accuracy and detection speed. ConNeXtV2 is then inserted into the backbone to enhance inter-channel feature competition. RepBlock and SimConv are selected to replace the original Conv and improve computational ability and memory bandwidth. For the loss function, CIoU is replaced by MPDIoU to ensure an efficient and accurate bounding box. Ablation tests show that our improved algorithm achieves better performance in all four metrics reflecting accuracy: precision, recall, F1, and mAP@50. Compared with the original model, whose four metrics are approximately 90%, the modified algorithm achieves above 95%. mAP@50 in particular reaches 95.6%, exhibiting an improvement of approximately 4.5%. Although complexity improves, the requirements of real-time fire and smoke monitoring are satisfied.

## 1. Introduction

The notion of Industry 4.0, often known as the Fourth Industrial Revolution, underscores the incorporation of digital technology into production operations. Smart factories use a combination of the Internet of Things (IoT), data analytics, and cloud computing to create networked and data-centric production processes. The advent of automation, artificial intelligence, robotics, and sensor technologies has led to significant advancements in manufacturing. These technological advancements facilitate the automation of monotonous jobs, augment accuracy, and amplify the overall productivity in factories. With ongoing technological advancements, factories are driven to maintain competitiveness by embracing the most recent developments. However, although innovations accelerate the development of relevant industries and stimulate the establishment of new factories, these new factories may lack mature regulations for safety production or face potential risks in production procedures. The repercussions of fires include several consequences, such as loss of human life, financial losses due to damage to buildings and residences, extensive social, health, and economic expenses resulting from evacuations, exposure to smoke, and a decline in tourist earnings [1,2,3]. According to a 2023 workplace safety and health report by the Ministry of Manpower in Singapore [4], the number of recorded occupational injuries, including mild injuries requiring medical absences or light tasks, amounted to 21,766. The number of major and fatal injuries reached 660, with approximately 80% of major injuries occurring in traditional industries, including construction, manufacturing, transportation, and storage. The total compensation amount for injured workers was USD 122.26 million, rising by 12% since 2021. Therefore, safe production is critical for factories to reduce their financial costs and protect their workers’ health. Fire is a significant factor in accidents during production. Almost all factories possess fire extinguishing equipment or have designed fire escape routes for evacuation; however, many fire accidents still occur every year. According to a recent report by the U.S. Bureau of Labor Statistics (BLS), fire ranks eighth among the top causes of work-related injuries, resulting in more than 1770 injuries in the U.S. On 22 September 2023, conflagration followed by successive detonations at a manufacturing facility for golf balls located in the southern region of Taiwan resulted in the death of nine individuals and caused injuries to over 100 others. On 21 November 2022, a fire broke out at a factory located in Wenfeng District, Anyang City, Henan, resulting in the unfortunate loss of 38 lives and injuries to two more individuals.

According to the report by the National Fire Protection Association (NAFA), between 2017 and 2021, fire departments in the United States dealt with an average of 36,784 fires per year at industrial or manufacturing buildings. The fires resulted in 22 fatalities, 211 injuries, and USD 1.5 billion in direct property damage each year, on average.

There are many traditional fire-monitoring methods, such as human inspection, sensors, and cameras. Employing human resources for fire monitoring offers several benefits, as experts in a certain region can discern slight alterations that may signify a heightened fire likelihood. In addition, human monitors can react promptly by notifying emergency services, mobilizing local firefighting resources, or even directly intervening to ensure safe conditions. However, there are limitations to human monitoring, such as limited coverage and visibility, inconsistent monitoring standards, and limited operational hours.

Sensor-based detection systems offer the advantages of continuous and consistent monitoring, data recording and analysis, real-time data collection, and quick alerts. Nevertheless, the expenses for the installation and maintenance of sensor-based systems can be substantial. This includes the costs associated with the sensors themselves, as well as any essential infrastructure, such as communication networks. A weather research and forecasting (WRF) model is an advanced detection model using sensors or detector data like daily temperature extremes, mean relative humidity, air and dewpoint temperature, and daily accumulated shortwave radiation [5,6]. For example, Nicole and Kumar, Mukesh, et al. succeeded in the simulation and prediction of the June 2005 fire weather for Interior Alaska and the October 2007 fire weather for California, respectively. However, it is mostly used in the area of wildfire detection rather than fire in cities. The factories may not have many detectors for humidity, radiation temperature, and so on. A wildland–urban interface fire dynamics simulator (WFDS) is another popular model for fire prediction; however, it is mostly used for predicting a fire’s rate of spread rather than detecting fire.

With the development of artificial intelligence, an increasing number of researchers have focused on the use of computer vision combined with deep learning to monitor fires in cities for early-stage detection. Image processing systems may provide higher cost efficiency than massive sensor networks or the constant use of human monitors, particularly when dealing with large regions [7]. Currently, most algorithms concentrate on forest fire detection [8,9,10,11,12,13] rather than factory fire detection. Fire detection studies are similar to factory fire detection and smart city fire detection [14,15,16,17]. Avazov et al. proposed a method based on the YOLOv4 network cooperating with the Banana Pi M3 board to detect fires in sunny and cloudy weather environments during the day and night [14]. Yar et al. integrated the stem module with a backbone, a P6 module in the head, and modified the larger kernels in the SSP [15]. The performance was also tested on a new medium-scale well-annotated dataset for fire detection in smart cities. Saydirasulovich et al. proposed a fire detection method based on YOLOv6 and created a dataset for smart cities collected from an Internet source, including 4000 images [16]. After detection, several machine-learning methods were used for multiclass object recognition, and XGBoost achieved the highest object identification accuracy. Talaat and ZainEldin presented an enhanced fire-detection method for smart cities using the YOLOv8 algorithm, referred to as a smart fire-detection system (SFDS) [17]. SFDS utilizes deep learning to identify fire-related characteristics in real-time. Although the above algorithms may exhibit good performances, the theme of smart cities is too narrow. After checking the datasets, images of traffic accidents, candles, or even BBQs were included, which are almost irrelevant to factories. Fire disasters in factories may have completely different backgrounds, resulting in low confidence in accuracy. To solve this problem and improve accuracy, we propose an improved fire detection model using computer vision and deep learning that specifies fire and smoke in smart factories. The specific work is as follows:

1. A dataset including more than 5000 images of factory fire and smoke is created and checked for similarities to ensure quality.
2. Each image in the dataset is labeled manually for smoke and fire with labeling tools.
3. ConNeXtV2 is used and inserted into the backbone part to enhance inter-channel feature competition for better feature mapping.
4. RepBlock and SimConv replace the original Conv and C2f parts to improve the computational ability and memory bandwidth.
5. The original CIoU is replaced by MPDIoU for an efficient and accurate bounding box.
6. After ablation, the modified algorithm achieves a significant increase in detection accuracy compared to the original algorithm for smart factories.

The remainder of this paper is organized as follows. Section 2 presents the most recent studies in the fields of machine and deep learning for fire and smoke detection. The proposed framework is presented in Section 3. Section 4 presents an experimental evaluation. Section 5 presents the results and discusses their implications. Finally, the conclusion is made in Section 6.

## 2. Literature Review

Fire and smoke detection techniques for visual identification may be categorized into two main categories: traditional detection systems that depend on image attributes combined with machine learning and detection methods that are based on deep-learning principles. Prior research in the domain of visual identification has mostly used feature extraction methods. Flame-specific chromatograms, flame motion, textures, shapes, and also lightness are all included. However, these methods have a complex and detailed process of manual feature extraction. Deep-learning algorithms are able to automatically extract complex information and characteristics from images, effectively solving the problems of duplication and interference that arise when humans extract visual data. Therefore, current academic inquiries have mostly concentrated on the use of deep-learning methods cooperating with computer vision for smoke or fire detection, and the results have consistently shown improved accuracy as well as decreased false fire and smoke alarm rates. 

### 2.1. Machine-Learning Method

For traditional fire detection methods, research has consistently concentrated on identifying the prominent characteristics of fire photographs using machine-learning techniques. Zhao et al. proposed a novel method using support vector machines (SVMs) to detect forest fires. This approach incorporated both static and dynamic information [18]. The fire-flickering frequency was determined by analyzing the Fourier descriptors of the flame contour using a temporal wavelet. This analysis is based on the fluctuation of the flame contour over a brief period. A total of 27 dynamic characteristics were used for the SVM-based classification. These features are calculated every 20 consecutive video frames. Foggia et al. presented a methodology for identifying fires by analyzing films captured by security cameras. Two primary innovations were implemented [19]. A multi-expert system combined complementary information derived from color, shape change, and motion analysis. Trung used a four-step method to detect fire. First, an adaptive Gaussian mixture model for detecting moving regions was used [20]. Then, a fuzzy c-means algorithm was used to segment candidate fire regions based on their color, followed by the extraction of special parameters that capture the tempo-spatial characteristics of the fire regions. Finally, an SVM algorithm showed the best performance in accurately distinguishing between fire and non-fire regions. Ko et al. used hierarchical Bayesian networks with intermediate nodes for fire detection. Four probability density functions were used to represent the evidence at each node. The probability density functions for each node were represented by the skewness of the red color and the three highest frequencies derived from a wavelet transform. The proposed system was successfully implemented in real-world contexts for various fire-detection tasks, efficiently differentiating fire from fire-colored moving objects [21]. Han et al. introduced a novel method for identifying fires in a video stream that maximized the use of the motion features and color information of a fire [22]. The first step involves motion detection using Gaussian mixture model-based background removal to extract moving items from a video feed. Subsequently, a multicolor-based detection method was used to identify potential fire areas by merging the RGB, HSI, and YUV color spaces. Ultimately, the two outcomes were merged to determine the precise locations affected by the fire. An innovative approach for smoke identification in videos that combines color and motion characteristics was introduced [23]. The outcome of the optical flow was presumed to approximate the motion field. The process of estimating the background and using a decision rule based on color was used to identify potential areas of smoke. The Luca–Kanade optical flow method was used to compute the optical flow at the selected locations. The motion characteristics were derived from the optical flow findings and used to distinguish smoke from other moving objects. Finally, a backpropagation neural network was used to categorize smoke as either fire-related or non-fire-related. Son et al. developed an automated color-model-based technique for detecting concrete structural components in color photographs with a high degree of accuracy using a machine-learning algorithm [24]. The RGB color space was converted to non-RGB color spaces to enhance the distinction between the concrete and background classes and to provide resistance to variations in lighting conditions. Subsequently, a comparative analysis was conducted on the performance of three machine-learning algorithms (Gaussian mixture, artificial neural network, and SVM models) in two non-RGB color spaces (HSI and normalized RGB).

### 2.2. Deep-Learning Method 

Deep learning has seen rapid development owing to advancements in hardware, such as GPUs and TPUs, together with parallel processing methods, enabling the training of larger and more complex neural networks within feasible timeframes. In addition, the presence of accessible deep-learning frameworks (such as TensorFlow and PyTorch) and tools streamlines the implementation and experimentation with deep learning. Developers may focus on creating architectures and optimizing parameters instead of becoming involved in the intricacies of low-level optimization and hardware management. These advancements have also expedited the use of deep-learning systems in the areas of computer vision, such as object detection, image classification, and image segmentation. Such developments also provide opportunities for researchers to use computer vision combined with deep-learning methods to propose improved deep-learning algorithms that specifically aim to detect fire or smoke for early alarms. There are many advanced and mature detection algorithms that can be simplified into two categories: one-stage detectors, including the single-shot multibox detector (SSD) [25], You Only Look Once (YOLO) [26] and its series, and RetinaNet [27]; and two-stage detectors, such as R-CNN [28], Fast R-CNN [29], Faster R-CNN [30], and Mask R-CNN [31].

The two-stage detection involves two main steps. The first step is the identification of possible zones of interest using various techniques, including selective search, edge boxes, and deep-learning-based approaches such as region proposal networks. The second step is to feed the proposed area into a classifier to ascertain the existence of items and enhance their localization. The classifier assigns class labels to every suggested area and adjusts the bounding boxes. Many researchers have focused on fire detection using two-stage detection algorithms. Chopde et al. presented a comprehensive monitoring system and forest fire detection model based on a fast R-CNN. The system is designed to identify forest fires by analyzing video frames taken by unmanned aerial vehicle drones [32]. Barmpoutis et al. [33] propose a fire detection method using Faster R-CNN. First, the potential candidate fire region was identified using Faster R-CNN, and then the prospective fire areas were mapped onto a Grassmannian space, where each picture was represented as a collection of points forming a cloud on the manifold. Finally, a vector representation method was used to combine the Grassmannian points by considering the criterion of proximity to the manifold. Zhang et al. proposed a novel approach called the multi-scale feature extraction model to enhance the performance of the traditional Faster RCNN target detection model for detecting small target forest fires [34]. The soft-NMS algorithm was used instead of the NMS method to minimize the loss of identified frames due to mistaken deletion. Pan et al. introduced an innovative framework for detecting and evaluating fire smoke in collaboration with weakly supervised fine segmentation and a lightweight Faster R-CNN [35]. A knowledge distillation technique and a three-input/one-output fuzzy system were used to reduce the complexity of the system and evaluate its severity level, respectively. 

Unlike two-stage detectors, a one-stage detector directly predicts the bounding boxes and class probabilities for multiple objects in a single pass through a network. One-stage detectors are known for their simplicity and efficiency, making them suitable for real-time applications. YOLO is a widely used deep-learning method for a range of computer vision applications, such as object detection in photos or videos. Its advantages include velocity and precision, making real-time fire detection possible and reducing false alarms. Former researchers focused on fire detection in smart cities, which is a large range compared to fires in factories. Fires in smart cities may involve different scenarios, such as fires in apartments, office buildings, and factories. Kuldoshbay et al. proposed a unique convolutional neural network to identify fire areas using the improved YOLOv4 network [14]. Automated color augmentation and parameter reduction were adopted to recognize and alert for the occurrence of catastrophic fires under various weather conditions. Yar et al. used the enhanced YOLOv5s model, which incorporated a stem module into the backbone, substituted larger kernels with smaller ones in the SPP (neck), and introduced the P6 module into the head to reduce the model size and complexity [15]. In addition, Saydirasulovich et al. tested the performance of YOLOv6 on a self-made dataset collected from website sources and proved its great potential for this task [16]. Random forests, k-nearest neighbors, SVMs, logistic regression, naïve Bayes, and XGBoost were used to assess the ability of the system to detect fire-related items. Talaat and ZainEldin proposed an enhanced fire-detection method for smart cities using the YOLOv8 algorithm, referred to as SFDS [17]. The smart city structure has four fundamental layers: application, fog, cloud, and IoT. After processing these four layers, the fire region in the image can be located, and the process is improved compared with the original algorithm. Sathishkumar et al. apply transfer learning to pre-trained models like VGG16, InceptionV3, and Xception to detect ongoing flames [11]. This approach enables researchers to use a smaller dataset and reduce computing complexity while maintaining accuracy. 

However, in different scenarios, fire and smoke have different backgrounds, and high detection accuracy and low false alarm rates can be achieved. Therefore, in this study, an algorithm aimed at detecting fires and smoke in smart factories was developed based on YOLOv8.

## 3. Proposed Framework

### 3.1. Model Structure of the YOLOv8n Network

YOLO series network is a widely used method in computer vision for real-time object recognition. It is renowned for its exceptional velocity and precision, making it a highly desirable option for several applications requiring instantaneous processing [36]. YOLO has undergone several modifications, including YOLOv3, YOLOv5, and YOLOv7, with each subsequent version resulting in improvements in both accuracy and speed. The most recent iteration, YOLOv8, incorporates the architectural improvements made in its previous versions, particularly YOLOv5 and YOLOv7. This system utilizes cutting-edge methods to extract features and train models, thereby guaranteeing its position as a leader in object identification technology. These technological developments enhanced the overall precision and resilience of the system [37]. The product line provides a selection of models of various sizes, including N/S/M/L/X scales, calibrated using scaling factors. 

**Figure 1:**
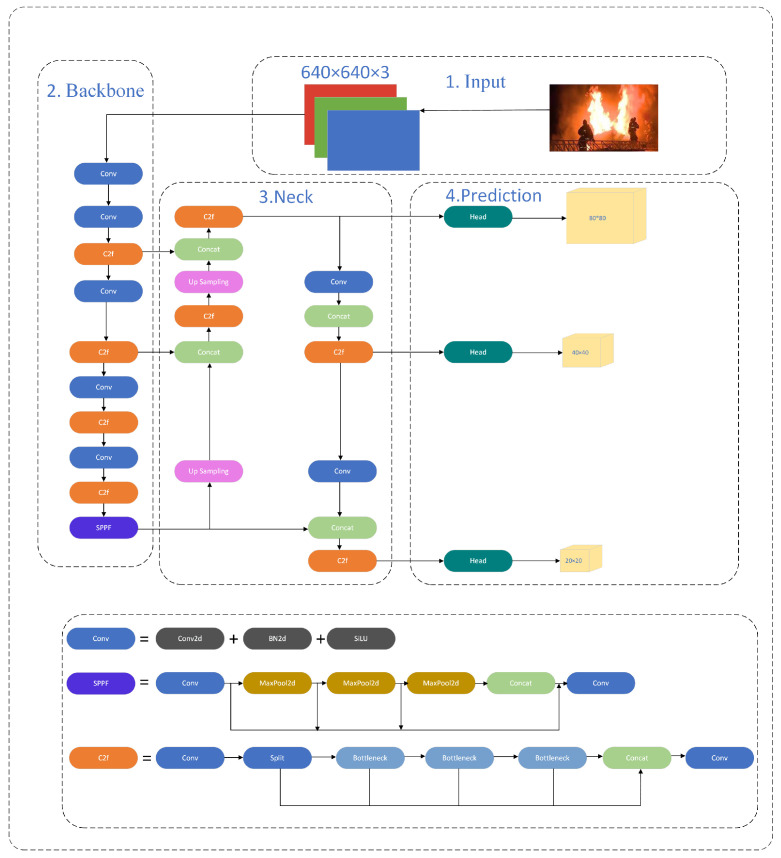
The structure of the YOLOv8n algorithm.

The head of YOLOv8 was extensively modified compared with the head of the former algorithm, including a decoupled structure that separated the classification and detection components, as shown in Figure 1. The YOLOv8 architecture is built around a series of consecutive convolutional layers that systematically extract significant information from the input picture. The YOLOv8 model’s architecture consists of many convolutional layers, which are then followed by fully connected layers. These layers are responsible for making predictions about the bounding boxes and class probabilities of the objects recognized in an image. In this study, loss computation uses the task-aligned assignment approach to allocate positive samples and distribute focal losses. Furthermore, the YOLOX model includes a data enhancement component that implements the mosaic enhancement technique in the last 10 epochs. This technique significantly improves model precision. YOLOv8 enhances the performance of its predecessors by introducing novel features and improvements. The product line has a range of versions of varying sizes, each of which includes unique design modifications to enhance performance and versatility. In this study, YOLOv8n is chosen from its series as it contains both fast detection speed, low complexity, and also high detection accuracy.

### 3.2. Using ConNeXt V2 to Enhance Inter-Channel Feature Competition 

ConvNeXt is a convolutional neural network (CNN) structure designed to overcome the drawbacks of conventional CNNs and enhance their performance to match those of vision transformers [38]. One distinguishing feature of ConvNeXt is its streamlined design. This simplifies the complexity of conventional CNNs while preserving their efficiency and efficacy. ConvNeXt is a scalable architecture that can be easily altered to different sizes and capacities, making it appropriate for a wide range of applications, from those that require limited computing resources to those that demand high-performance models. The ConvNeXt model outperforms the Swin Transformer in terms of COCO detection and ADE20K, two famous and significant datasets used in computer vision, offering a consistent structure for creating and evaluating object-identification algorithms. The ConvNeXt model utilizes a 4 × 4 convolutional kernel with the same stride as that of the Swin Transformer system to downsample feature maps, leading to a marginal increase in accuracy. Multiple sizes of convolutional kernels were tested in the ConvNeXt model, and the findings suggested that the 7 × 7 kernel yielded optimal performance and maximum accuracy.

**Figure 2:**
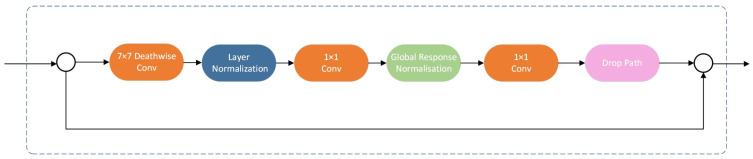
The architecture of ConvNeXt V2.

CNNs, such as ConvNeXt, have shown exceptional performance in many situations owing to ongoing improvements in representation-learning frameworks and topologies. However, ConvNeXt can be modified to be lighter and faster. Researchers have attempted to integrate ConvNeXt with self-supervised learning approaches such as masked autoencoders, although performance was deemed inadequate. Thus, a fully connected multi-attention embedding and graph reasoning network (GRN) layer was added to the architecture of ConvNeXt V1 to amplify the rivalry among channels for better feature representation. The newly formed model, ConvNeXt V2, combines self-supervised learning approaches and architectural enhancements. The block architectures of ConvNeXt V2 are displayed in Figure 2. In ConvNeXt V2, the GRN layer was included after the MLP layer; however, the superfluous LayerScale was removed, distinguishing it from ConvNeXt V1. There are three steps in the procedure of GRN: (1) global feature aggregation, (2) feature normalization, and (3) feature calibration, which helps the entire model increase the contrast and selectivity of the channels. Compared with the other three widely used normalization layers, local response normalization [39], batch normalization [40], and layer normalization [41], GRN performs better in a supervised baseline. In conclusion, the ConvNeXt V2 model incorporated a fully convolutional masked autoencoder architecture and introduced a new global response normalization layer. This combination enhanced the effectiveness of mask-based self-supervised learning and demonstrated excellent performance in object identification.

### 3.3. Using RepBlock and SimConv to Improve the Computational Ability and Memory Bandwidth 

**Figure 3:**
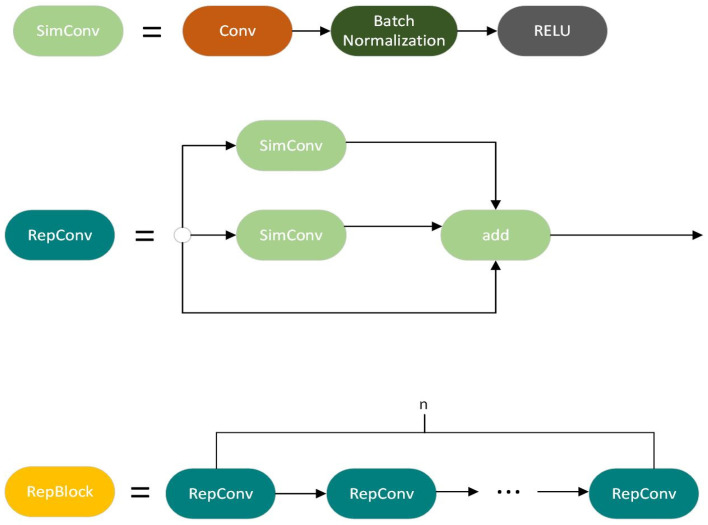
The architecture of Simconv, RepConv, and RepBlock.

Many classical algorithms, such as Inception and ResNet, have been proposed for image classification and object detection. Although these well-designed algorithms have succeeded in various vision tasks, they do not achieve a suitable and efficient accuracy-speed balance on the deployed hardware. Research has been conducted on the design and implementation of deep-learning networks for optimal hardware efficiency [42,43]. An important problem is the creation of a hardware-friendly network with fast detection speed and high detection accuracy. Repconv utilizes two different sizes of branches: 3 × 3 Simconv and 1 × 1 Simconv, which can improve the efficiency of utilizing the computational ability of hardware. The difference between Simconv and Conv lies in their activation functions. ReLu was used in Simconv rather than Silu in Conv. The ReLU function is computationally simple compared with the SiLU function, which enables rapid computation and is advantageous in both the training and inference of neural networks. The ReLU addresses the issue of the vanishing gradient problem, which is often encountered when using activation functions such as a sigmoid or tanh, and offers a more effective solution than typical sigmoid functions. However, in some instances, it is not as efficient as ReLU because it has nonzero gradients for negative inputs. In addition, ReLU is advantageous because of its simplicity, which makes it more straightforward to implement and less susceptible to errors or numerical complications than SiLU. This makes it a preferred option for novices or when engaging in quick prototyping. Thus, because of its efficient computing, simplicity, and sparse activation, ReLU is often favored, particularly in situations with limited computing resources or when dealing with complex networks. The advantages of ReLU can also be observed in Simconv because of the replacement of SiLU by ReLU, and also in Repblock and RepConv, because of their use of Simconv. The structures of Simconv, RepBlock, and RepConv are shown in Figure 3. 

### 3.4. MPDIoU: Loss for Efficient and Accurate Bounding Box Regression

The utilization of the anchor-free concept resulted in significant modifications to the loss function employed in YOLOv8, distinguishing it from YOLOv5. The optimization direction comprises two distinct components: classification and regression. The classification loss continues to employ binary cross-entropy loss, whereas the regression component incorporates the distribution focal loss (DFL) and bounding box regression loss. The comprehensive loss function can be formulated as

(1) $$f_{loss}=\lambda_{1}f_{BCEL}+\lambda_{2}f_{DFL}{+\lambda }_{3}f_{BBRL}.$$

One of the categories of loss in prediction is the cross-entropy loss, which can be expressed as

(2) $$f_{BCEL}=weight\left[class\right](-x\left[class\right]+log(\sum_{j}exp(x\left[j\right]))).$$

In this context, “*class*” represents the total number of categories. “*weight* [*class*]” refers to the weights assigned to each class. Lastly, “*x*” represents the probability value resulting from the sigmoid activation function. The DFL optimizes the focal loss function by extending the discrete outcomes of classification to continuous outcomes through the process of integration. The equation is as follows:

(3) $$f_{DFL}\left(S_{i},S_{i+1}\right)=-(\left(y_{i+1}-y\right)\log\left(S_{i}\right)+\left(y-y_{i}\right)\log\left(S_{i+1}\right).$$

The utilization of a loss function in bounding box regression is crucial in the context of object detection because it can significantly enhance the performance of the model. Most prior research assumes that the training data comprise high-quality examples and prioritizes enhancing the fitting capability of the bounding box regression losses; in the original YOLOv8 network, the border regression loss utilizes the complete intersection over union (CIoU) metric. However, the loss function of the CIOU has the disadvantage of low detection accuracy during the training process. The MPDIoU loss function provides a potentially better solution than CIoU, which provides a more precise method for evaluating bounding boxes compared to the original CIoU loss function. This leads to an enhanced detection performance and accuracy during training and optimization.

**Figure 4:**
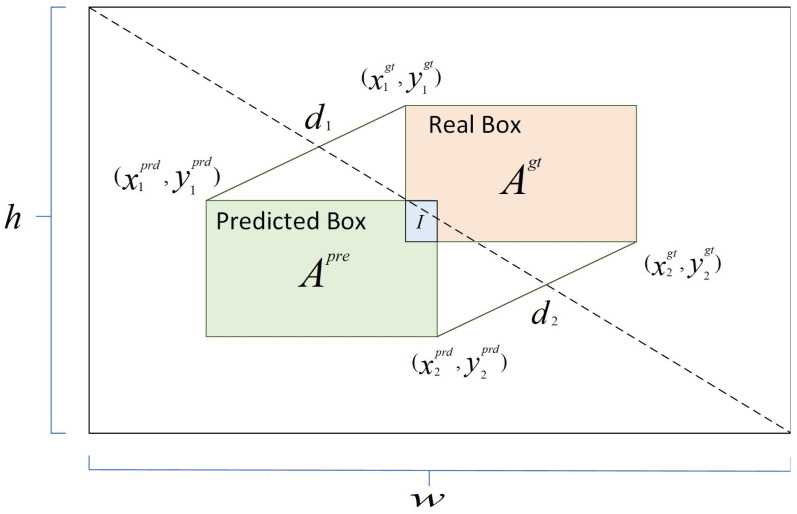
Schematic diagram of MPDIoU.

The calculation procedure for MPDIoU is described below. Given two arbitrary convexes, A and B, which are the real and predicted boxes of the object detection process, the calculation and definition of the parameters are shown in Figure 4. Variables *w* and *h* represent the width and height of the input image, respectively. ${(x}_{1}^{prd},y_{1}^{prd}),\left(x_{2}^{prd},y_{2}^{prd}\right),\left(x_{1}^{gt},y_{1}^{gt}\right),and\;\left(x_{2}^{gt},y_{2}^{gt}\right)$ are used to represent the upper-left and lower-right corner points of polygons *A^Prd^* and *A^gt^*, respectively.

(4) $$d_{1}^{2}={\left(x_{1}^{prd}-x_{1}^{gt}\right)}^{2}+{\left(y_{1}^{prd}-y_{1}^{gt}\right)}^{2},$$

(5) $$d_{2}^{2}={\left(x_{2}^{prd}-x_{2}^{gt}\right)}^{2}+{\left(y_{2}^{prd}-y_{2}^{gt}\right)}^{2},$$

(6) $$MPDIoU=\frac{I}{A^{gt}+A^{prd}+I}-\frac{d_{1}^{2}}{w^{2}+h^{2}}-\frac{d_{2}^{2}}{w^{2}+h^{2}},$$

(7) $$L_{MPDIoU}=1-MPDIoU.$$

### 3.5. Structure of Improved YOLOv8n for Factories Fire and Smoke Detection

**Figure 5:**
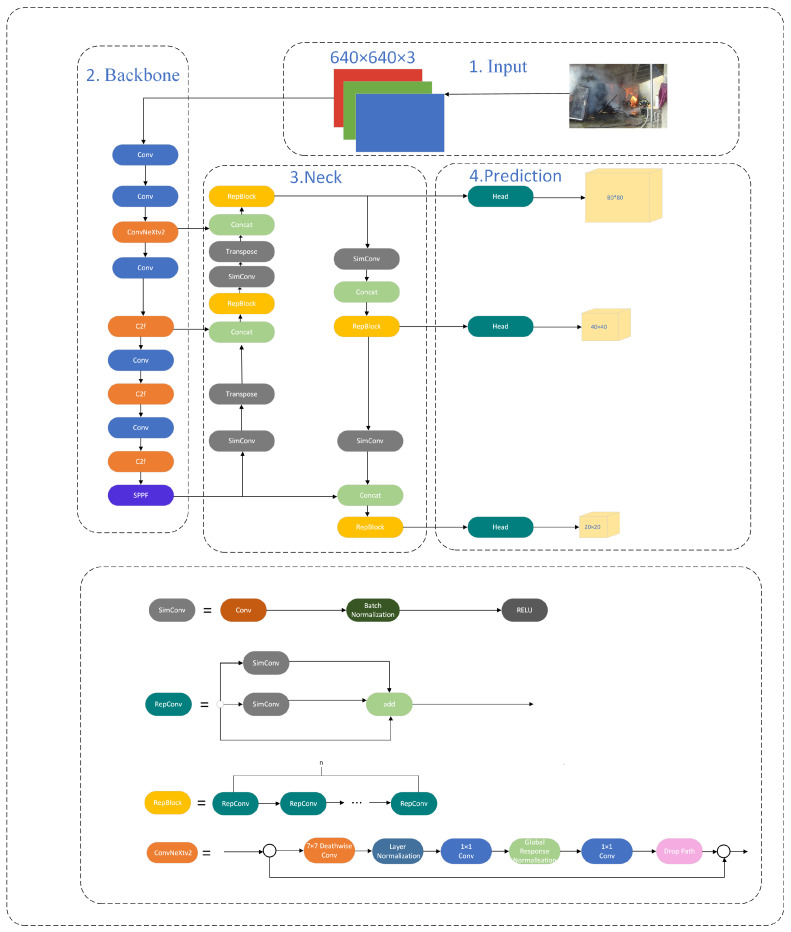
The structure of the improved YOLOv8n for factory fire and smoke detection.

After all these improvements in the backbone part with ConvNeXt V2, the neck part with SimConv and RepBlock, and the loss function with MPDIoU, a new modified algorithm aimed at detecting fire and smoke is formed, and its structure is shown in Figure 5. The performance is tested using self-made and self-labeled datasets. 

## 4. Data Collection and Experiment Setting

### 4.1. Datasets 

**Figure 6:**
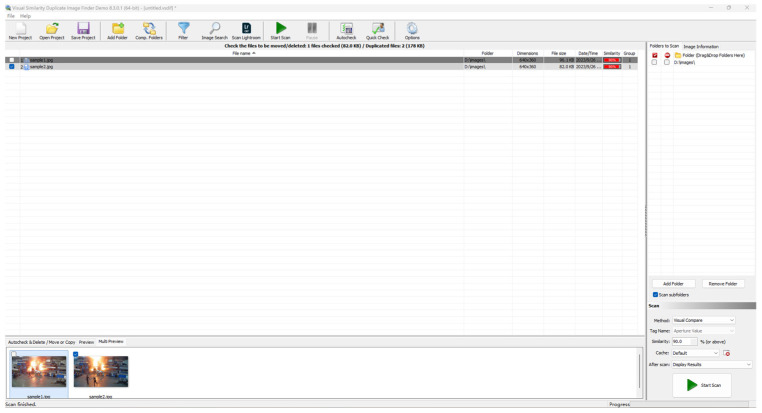
The after-process after collection of images using Visual Similarity Duplicate Image Finder.

Fire disasters can occur inside factories, such as production areas, loading docks, and offices. When a fire occurs in these areas, the background and brightness differ from those in areas outside the factories, such as receiving areas, shipping areas, and car parks. Therefore, to better simulate and detect fire disasters in factories, two types of datasets are considered: images of fire taken inside factories’ rooms and images of fire taken outside of factories’ rooms. When a fire disaster happens inside factories, the first reaction of workers and staff is to extinguish the fire or escape from the fire site when control is lost. However, when a fire occurs outside factory buildings, passers-by may take videos or images and enlarge the dataset of fires outside factories. The foundation of the dataset comprises searching images of two categories of factory fires and using labeling tools to locate the positions of fire or smoke in the collected images. This task was performed by search engines such as Baidu and Google, which searched for keywords such as “factory + fire, factory + fire + inside and factory + smoke”, and “factory + fire + outside”. Python was then used as a web crawler to efficiently retrieve the photos. Each image was thoroughly examined for copyright infringement, specifically for academic purposes. An issue encountered in the process of gathering datasets is picture duplication, in which several websites may have identical photos collected by web crawlers. The variety of the dataset was enhanced by preprocessing all photos before labeling them using the Visual Similarity Duplicate Image Finder (Figure 6; v. 8.3.0.1). This program adjusts the similarity score on a scale from 0 to 100%. Additionally, it can scan entire folders to identify and compile a list of similar photographs. Images with a similarity exceeding 90% were considered duplicates and were removed to maintain the quality of the datasets. The procedure of checking similarities makes sure similar images are not included in datasets, thus ensuring that the algorithm behaves well in training and also has good performance in real-time smoke and fire detection in factories.

**Figure 7:**
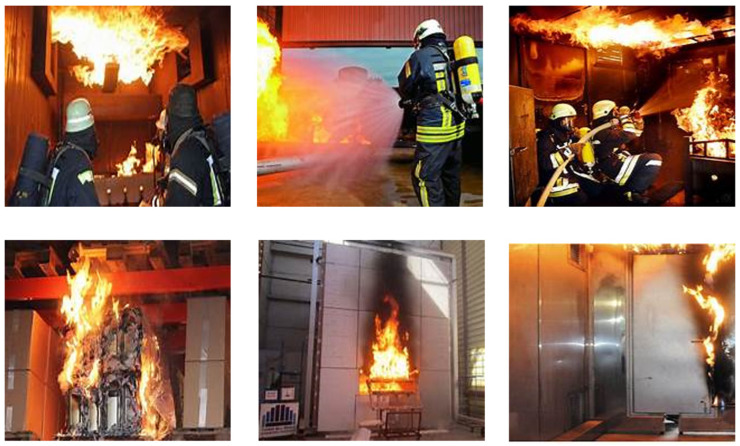
Examples of factory fire disaster images inside.

**Figure 8:**
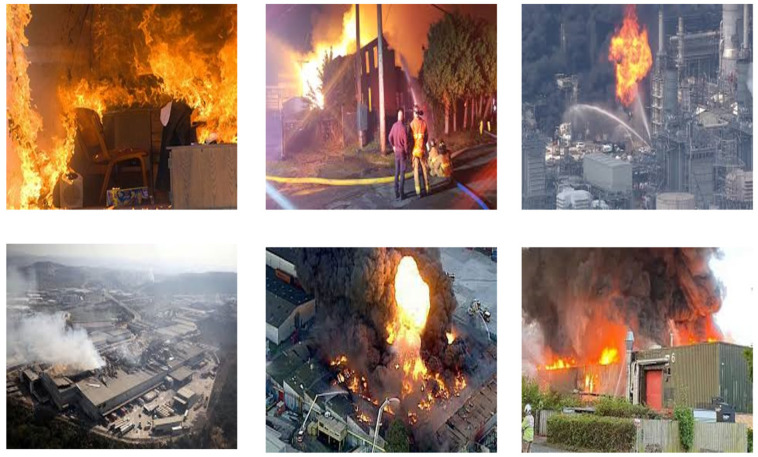
Examples of factory fire disaster images outside.

After these processes, 5002 images of factory fires were selected to form a factory fire dataset (including 1599 inside room and 3403 outside room fire disaster images). Samples of the fire and smoke inside and outside the factory rooms are shown in Figure 7 and Figure 8, respectively. The division of the dataset into two categories ensures the algorithm can be used at all angles and everywhere to detect fire and smoke.

Then, researchers used Labellmg as a labeling tool to label fire and smoke in the images of clean and unduplicated datasets. LabelImg is a widely used graphical application for annotating pictures by labeling objects and is often used for computer vision tasks. This is particularly advantageous for generating datasets to train machine-learning models. It is compatible with diverse formats, user-friendly interfaces, and cross-platform outputs. All labels were generated in the VOC format. Academics created and positioned the labels to ensure that the topic and substance of the photographs accurately represented the fires. A one-to-one correlation was established between the labels and images, with each image containing an associated label. Compared with the other datasets on fire, the newly formed dataset narrows the theme of images from fire in smart cities toward fires in factories. Researchers have also focused on smoke as a derivative of fire. Observing not only fire but also smoke can improve the detection efficiency and reduce the false alarm rate. All datasets were uploaded to Google Drive (https://drive.google.com/drive/folders/1xnZX_fZ6_QU-J1zDmI-AMm07kvAmIuvN?usp=sharing; accessed on 12 June 2024). The photographs and labels were sequentially numbered and organized into separate subfolders labeled “inside” and “outside”.

**Figure 9:**
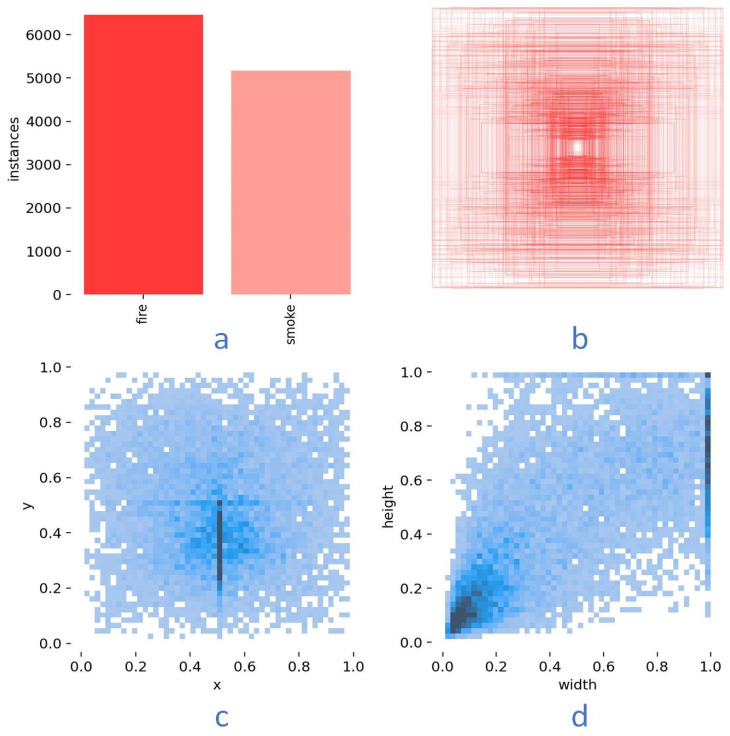
Visualization results of the self-made datasets and labels. (**a**) The number of labels for fire and smoke labels; (**b**) the size of the labels; (**c**) the distribution of labels’ centroid locations of the total image; (**d**) the distribution of labels’ size of the total image.

After the collection and formation of the images and corresponding labels, the dataset was divided into three parts: 70% for training, 20% for testing, and 10% for validation, as shown in Table 1. The division of the dataset can ensure the algorithm overfitting and good performance in new raw data. Figure 9 displays the visualization outcomes of the dataset analysis. Figure 9a shows that more than 6000 fire labels and more than 4000 smoke labels were formed. More than 10,000 labels ensure the dataset quality. Figure 9b shows the size labels; different sizes of fire and smoke in the images were considered and gathered. Thus, even a small size of fire and smoke will not be ignored. Figure 9c displays the distribution of the locations of the object’s centroid, with the horizontal and vertical coordinates denoting the centroid positions. Researchers have considered almost all fires and smoke at different positions. Figure 9d depicts the distribution of the item sizes, where the horizontal and vertical axes mean the width and height of the fire or smoke labels, respectively. Large fires and smoke are easily detected; therefore, even small size of fires and smoke in images are the most popular and considered. 

### 4.2. Experimental Environment 

The investigation was conducted using a Windows 11 operating system with an 11th Generation Intel i9-11950H central processor unit and an RTX A3080 graphics processing unit from Intel, Singapore. The GPU acceleration environment was created using CUDA 11.3, whereas the network architecture was built using Python 3.9 and PyTorch 1.11.1. The study used Visual Studio Code version 1.75.0, the details of which are listed in Table 2.

### 4.3. Hyperparameter Setting

All the experiments were performed using the hyperparameters listed in Table 3 to demonstrate the effectiveness of the proposed method. The 300 epochs ensure the algorithm is well-trained. 

### 4.4. Model Evaluation Metrics

The performance of the constructed model was evaluated using the following metrics: precision, recall, F1-score, and mAP. The metrics are defined as follows:

(8) $$P(Precision)=\frac{TP}{TP+FP},$$

(9) $$R\left(Recall\right)=\frac{TP}{TP+FN},$$

(10) $$F_{1}=2\times \frac{Precison\times Recall\;}{Precison+Recall},$$

(11) $$AP=\int_{0}^{1}P\left(R\right)dR,$$

(12) $$mAP=\frac{1}{N}\sum_{i=1}^{N}AP_{i}$$

The precision (P) rate refers to the ratio of targets correctly identified by the model to the total number of targets detected. Equation (8) is the mathematical expression used to compute the accuracy rate. TP denotes the precise anticipation of fire or smoke, whereas FP represents an incorrect forecast of fire or smoke in a factory. Recall (R) indicates the proportion of targets correctly predicted by the model as a percentage of all targets. The formula for calculating the recall rate is shown in Equation (9). FN indicates that the target is fire or smoke, whereas the algorithm considers it to be in a normal state. The average precision (AP) is the integral of the precision–recall curve, which is the area under the curve. When extra boxes are included, the precision value is shown using a precision–recall curve, indicating a greater recall value resulting from a lower class probability threshold. A robust model can maintain a high level of accuracy when the retrieval increases [34]. Usually, the threshold for intersection over union is set to 0.5. Typically, a high AP value indicates superior model performance. mAP@0.5 is the average of the APs for all target categories. The equations for computing the AP and mean average precision (mAP) are presented in Equations (11) and (12), respectively.

In addition to accuracy, complexity was considered using characteristics such as parameters, FLOPs, and FPS. The total number of parameters in a CNN is a critical determinant that substantially affects the complexity, memory requirements, and training duration of the model. Models with greater complexity, characterized by a higher number of layers and neurons, generally possess a larger parameter count, resulting in longer training durations. FLOPs refer to the examination of floating-point operations in CNNs as a part of academic research. This entails analyzing the computational requirements associated with the various layers and operations. The abbreviation “FPS” stands for the frequency at which frames, or visual representations, are processed or generated inside a CNN model or computer vision system. The FPS metric has immense importance in the field of CNNs, as it serves as a critical indicator for evaluating the computational efficiency and real-time capacities of tasks associated with image or video processing. These evaluations are valuable for evaluating the performance of fire networks.

## 5. Discussion 

### 5.1. Base Algorithms Selection

This study aims to develop an advanced system for factory fire detection. Therefore, it is important to find the most suitable algorithms and make improvements based on their disadvantages and detection tasks. Nine well-established object detection algorithms were selected from the literature: Shufflenet v2 [44], Faster R-CNN ResNet50 [45], SSD Inception_V2 [46], SSD MobileNet_V1 [47], DenseNet [48], swin transformer [49], YOLOv3-tiny [26], YOLOv5s [50], YOLOv7-tiny [51], and YOLOv8n [52]. These advanced algorithms were specifically trained for fire and smoke detection using self-made factory fire datasets. The basic parameters of these studies and their corresponding smoke and fire detection results are summarized and listed in Table 4.

YOLOv7 exhibits the highest value of precision, reaching 0.924, which proves its good detection of smoke and fire in factories. However, YOLOv7-tiny falls behind YOLOv8n in terms of Recall, F1, and mAP @50. In fact, YOLOv8n has recall, F1, and mAP @50 of 0.917, 0.920, and 0.911, respectively, and ranks first among all the models. Therefore, YOLOv8n shows great performance in detection accuracy among the selected advanced models. However, accuracy is only one part of the detection process, while complexity influences the detection speed. FLOPS and FPS were used to represent the complexity of the algorithms. After observation, Inception_V2 had the smallest FLOPS, indicating that floating-point operations were minimal. However, lower FLOPS do not imply fewer parameters or a higher FPS. Comprehensive optimization is also a major issue in algorithms. YOLOv8n has the least number of parameters, which is only 3.01 million. The second-lowest quantity, MbileNet_V1, is approximately half. YOLOv8n has the highest FPS, which means that it can reduce the detection time and provide an early response when a fire occurs. The empirical results for nine commonly used detection methods demonstrate that YOLOv8n offers significant advantages in terms of both precision and efficiency. Consequently, YOLOv8n is selected as the primary algorithm for further advancement and refinement.

### 5.2. Ablation Tests Results

Simconv, Repblock, and ConNeXtV2 were selected to insert or replace Conv and C2f in the neck and backbone parts of the original YOLOv8 because of their excellent characteristics in feature extraction. In addition, MPDIoU replaced CIoU in the loss function. Ablation tests were performed to validate the improvements; the results are shown in Table 5.

**Figure 10:**
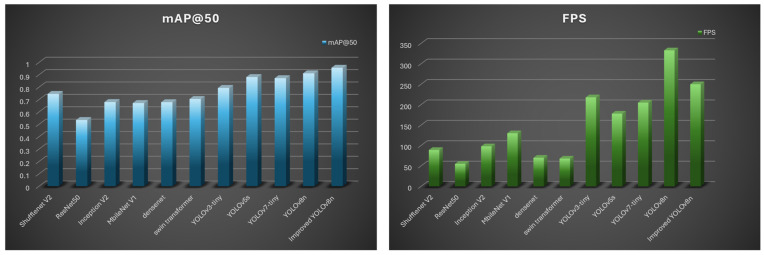
Improved YOLOv8 vs. the other methods: bar charts of FPS and mAP@0.5.

The first row in Table 5 displays the outcomes of the original YOLOv8n network without any enhancements that served as a baseline for the comparison. The precision, recall, F1, and mAP @0.5 of the three new models (rows 2–4) increase compared to the previous ones, which proves the efficiency of the replacement. In particular, when SimConv and RepBlock were used, the precision and recall reached 0.931 and 0.932, respectively. Subsequently, three different combinations were formed and tested (rows 5–7), and the results show better performance than that of the former three models. Finally, SimConv, Repblock, ConNexTV2, and MPDIoU were used in the proposed modified algorithm, achieving excellent accuracy. The precision, recall, and F1 significantly increased, exceeding 0.95. mAP@0.5 improved most significantly, increasing by 4.5% from 91.1% to 95.6%. Although the complexity of the algorithm increases to a certain extent, the consequences of the FPS are maintained at 250, which is sufficient for real-time fire monitoring in factories. This is also acceptable for a large increase in the detection accuracy with a small decrease in the detection speed. The comparison of mAP@50 and FPS for the improved YOLOv8n, original YOLOv8n, and the other advanced method is shown in Figure 10. It can be observed that the improved model has the highest score of mAP@50 with only a small decrease in FPS. Its FPS still ranks second among all models.

**Figure 11:**
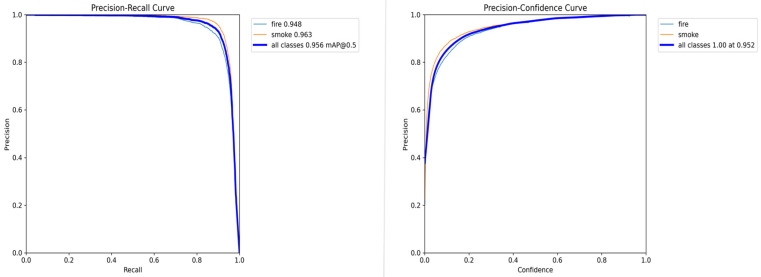
Precision-recall curve and precision-confidence curve.

**Figure 12:**
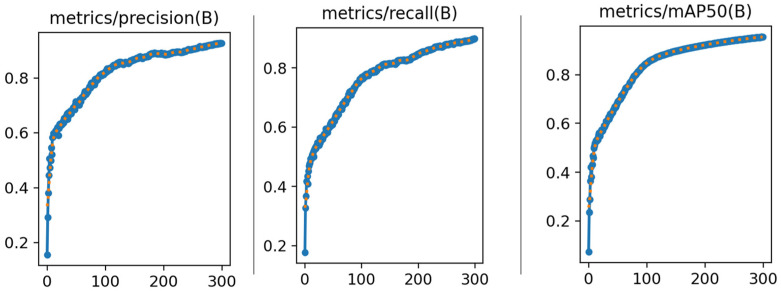
The curve of precision–epochs, recall–epochs, and mAP–epochs.

Precision–recall and precision–confidence curves are shown in Figure 11. After the training process, the improved model achieved high detection accuracy for fire and smoke of 0.948 and 0.963, respectively. Besides that, all classes achieve 0.956 for mAP@0.5, which is a quite high score and can satisfy the need for fire disaster detection for smart factories. The changes in the precision, recall, and mAP50 with an increasing number of epochs are shown in Figure 12. The precision, recall, and mAP50 all possess a significant increasing trend in the beginning training process and tend to be steady in the end, indicating that the model has been successfully trained. 

**Figure 13:**
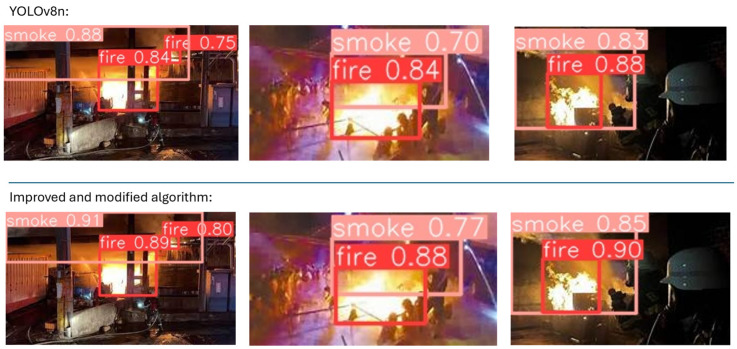
Visible experiments of improved and original algorithms for various indoor environments in factories.

**Figure 14:**
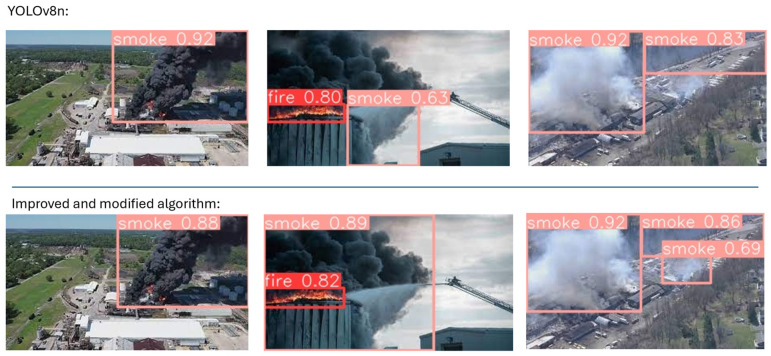
Visible experiments of improved and original algorithms for various outdoor environments in factories.

This system was also tested for its performance in real-world scenarios using both images of fires occurring inside a factory and fires occurring inside, but for which control was lost and the fire expanded outside the factory. Figure 13 and Figure 14 illustrate fire detection consequences under different conditions.

Figure 13 and Figure 14 show that the confidence for both fire and smoke was improved by the enhanced algorithm for images taken both inside and outside the factory. In addition, in the last comparison images, the modified algorithm detects more smoke than in the original, which proves that the modified algorithm can detect even a small fire signal. Thus, the modified algorithm can be used in camera and edge devices at different angles. 

### 5.3. Discussions and Limitations 

The success of the unique system may be attributed to the capability of the YOLOv8n algorithm to accurately detect and locate flames and smoke both inside and outside factory rooms, which ensures that the camera settles both inside and outside of the factory. Several factors, including fluctuations in illumination, the presence of smoke, changes in sensitive environmental elements, and obstacles, may influence the performance of the detection model. The categorization of self-made datasets and the labeling tasks of fire and smoke were crucial for achieving this goal and ensuring the successful implementation of the algorithm for fire and smoke detection following the training phase. Although the performance of the original algorithm was good, the detection accuracy could still be improved. Even small improvements in the accuracy of fire detection algorithms may help workers escape from fire disasters or firefighters arrive at the scene early, which helps to protect human lives and property. To further improve the accuracy of the algorithm, we used ConvNeXt V2 to enhance the inter-channel feature competition; thus, more representative features can be selected. RepBlock and SimConv were then selected to improve the computational ability and memory bandwidth and replace parts of C2f and Conv in the neck. Finally, the loss function was also improved and modified using MPDIoU for its efficiency and accuracy. Ablation tests showed that the improved algorithms improved the four metrics (precision, recall, F1, and mAP @50), reflecting an accuracy increase from approximately 90% to above 95%. The improved algorithm not only improves confidence in detecting fire and smoke but also detects more fire and smoke than the original algorithm. In the initial state of a fire disaster, the fire may easily be occluded, making it harder to detect fire in the early stages. The detection of a fire’s important derivative smoke can be a good supplement detection method when occlusion occurs. Smoke possesses different colors and textures than fire and may rise to a higher place to avoid occlusion. The brightness of the fire disaster and smoke within the room is lower in comparison to the fire disaster occurring outside the room. Under some circumstances, the level of light within the dataset may be quite low, while the level of brightness outside can be very high. Following the testing, the enhanced model demonstrates favorable detection outcomes on the dataset, indicating its ability to handle environments with varying levels of brightness. In conclusion, the proposed algorithm achieves excellent performance in detecting factory fires using deep learning and computer vision. 

Nevertheless, our dataset and technique have certain limitations. Initially, our research concentrated on the existence or nonexistence of factory fires without considering their extent and progression. In addition, after the improvements, the parameters and FLOPs increased to a small extent, which means that the improved algorithm has higher complexity and more calculations. Moreover, implementing the improved model may also need the cooperation of edge devices like sensors, cameras, computers, and so on. With the rising number of edge devices, ensuring performance and reliability becomes more difficult. 

To address these constraints, we should not only concentrate on the incidence of a fire but also carry out thorough studies of the aftermath, such as studying the development of the fire in the factory, evaluating the workers’ escape paths, and helping them find the fastest routes. Furthermore, other well-established methodologies and algorithms for optimizing the performance of the model can be gathered and used in cooperation with the improved algorithm. For example, transformers are commonly used in the fields of natural language processing and computer vision. The temporal difference method, which is commonly used in the area of moving-object identification and tracking research, can also be included in research on fire-escape route planning in factories. Regarding algorithm complexity, after our experiments, FPS still exceeded 200, which can also satisfy the need for real-time fire detection for the factory. In future studies, a more lightweight self-attention mechanism or convolution such as GhostNetv2 [53] or MobileNetV3 [54] will be tried to cooperate with the improved to make it lighter and faster. For the concern of edge devices, more tests will be conducted using different brands and types of edge devices to make the algorithm more suitable for smart factories. 

## 6. Conclusions 

In this paper, an improved deep learning algorithm based on YOLOv8n for smoke and flame detection during fire disasters in factories was proposed. Although there are many fire-detection algorithms or systems for forests or cities, our proposed model may be the first fire-detection method designed for factories. Through comprehensive trials and rigorous system assessments, it was shown that the proposed system is highly effective in promptly detecting flames and smoke in difficult environments. A dataset had to be created first because there is no existing dataset for factory flames and smoke. After searching and checking their similarities, 1599 images of fires inside factories and 3403 images of fires outside factories were selected and labeled to form the flame and fire tasks. Subsequently, studies on deep learning and object detection were researched, and many algorithms, such as ResNet50, DenseNet, and YOLO series, and their performances were tested and recorded on our self-made dataset. Precision, recall, F1, and mAP @50 were selected to represent accuracy, whereas the parameters FLOPs and FPS were used to represent speed. The experimental results show that YOLOv8n has the best performance in terms of both parameters and speed. We then attempted to improve its performance using the ConNext V2 inter-channel feature competition and abandoned abundant features. Subsequently, RepBlock and SimConv were selected and inserted into the modified model to improve the computational abilities and memory bandwidth. Finally, the original CIOU was replaced by MPDIoU to create a more efficient and accurate bounding box. After the former improvements and modifications, ablation tests were performed, and their consequences were compared. The results showed that compared with the original YOLOv8 model, the precision of the improved model increased from 92.2% to 95.3%, recall increased from 91.7 to 95.6%, F1 increased from 91.9% to 95.4%, and mAP@0.5 increased from 91.1% to 95.6%. All four metrics reflecting the accuracy of the improved algorithm exceeded 95%. Among these metrics, mAP @0.5 exhibited the highest improvement, increasing by approximately 4.5%. A limitation of our algorithm is that its complexity increases slightly after improvements; however, FPS does not drop significantly and can still exceed 250, which satisfies the requirements of real-time fire detection for smart factories. 

## Figures and Tables

**Table 1:** Distribution of fire and smoke images in the collecting dataset.

Dataset	Training Images	Testing Images	Validation Images	Total
Photos taken inside factory rooms	1119	320	160	1599
Photos taken outside factory rooms	2382	681	340	3403

**Table 2:** The environment of hardware and software for training.

Schedule	Capacity
Parameters configuration	Windows 11
CPU	11th Gen Intel(R) Core(TM) i9-11950H @ 2.60 GHz
GPU	RTX3080
RAM	128 GB
Deployment environment	Python 3.9.10
Deep-learning framework	Pytorch
Accelerated computing architecture	CUDA 11.3
Development platform	Visual Studio Code 1.75.0

**Table 3:** Improved model training hyperparameters for factory fire and smoke detection.

Training Parameters	Details
Epochs	300
Batch size	16
Image size (pixels)	640
Initial learning rate	0.01
Optimization algorithm	SGD
Pre-training weights	None

**Table 4:** Comparing the classical deep learning algorithms based on the dataset.

Models	Precision	Recall	F1	mAP@50	Parameters (m)	FLOPs (G)	FPS
Shufflenet V2	0.733	0.781	0.756	0.745	34.2	41.8	89
ResNet50	0.563	0.556	0.559	0.534	42.18	428.8	55
Inception V2	0.703	0.712	0.707	0.679	13.46	7.7	98
MbileNet V1	0.689	0.691	0.690	0.671	5.65	12.9	130
densenet	0.697	0.671	0.684	0.678	66.84	32.6	70
swin transformer	0.764	0.705	0.733	0.704	28.14	46.9	68
YOLOv3-tiny	0.812	0.803	0.807	0.793	8.725	12.8	218
YOLOv5s	0.902	0.883	0.892	0.881	7.02	15.9	178
YOLOv7-tiny	0.924	0.874	0.880	0.872	6.14	13.2	205
YOLOv8n	0.923	0.917	0.920	0.911	3.01	8.2	333

**Table 5:** Results of ablation experiments of usage of ConNeXtV2, Simconv/Repblock, and MPDIoU.

Models	Precision	Recall	F1	mAP@0.5	Parameters (m)	FLOPS (G)	FPS
YOLOv8n	0.922	0.917	0.919	0.911	3.01	8.4	333
YOLOv8n + Simconv/Repblock	0.927	0.928	0.927	0.915	3.62	12.3	270
YOLOv8n + MPDIoU	0.924	0.925	0.924	0.914	2.72	8.4	333
YOLOv8n + ConNeXtV2	0.922	0.921	0.921	0.919	3	9.3	285
YOLOv8n + Simconv/Repblock + MPDIoU	0.931	0.929	0.930	0.932	2.71	12.3	270
YOLOv8n + Simconv/Repblock + ConNeXtV2	0.936	0.938	0.937	0.936	2.71	13.2	250
YOLOv8n + ConNexTV2 + MPDIoU	0.928	0.934	0.931	0.931	2.99	9.3	285
YOLOv8n + Simconv/Repblock + ConNexTV2 + MPDIoU	0.952	0.956	0.954	0.956	3.81	13.2	250

## Data Availability

Data are contained within the article.
